# Public health in South Africa 1975–89: reflections on a momentous past

**DOI:** 10.3402/gha.v6i0.20095

**Published:** 2013-01-24

**Authors:** John Gear

**Affiliations:** Inaugural chair of the Department of Community Health, University of the Witwatersrand

It is an honour for me to reflect on a momentous period in the evolution of South Africa's Public Health. In 1979, Wits University appointed its first full-time Chair of Community Health to a post, which had been approved four years earlier. The delay in filling the post was a product of bureaucratic challenge and uncertainty around the job description of the incumbent. The bureaucratic challenge centred on the post being a tri-partite appointment, with the successful candidate answerable to the National Department of Health, the Provincial Department of Hospital Services, and the University of the Witwatersrand. A further complication was that most public health activities took place under the auspices of the Johannesburg Municipality which was not a part of the tri-partite arrangement but which had a strong presence on the selection committee.

There was much confusion back then as to what constituted community health, or public health, as it had been previously named and is now renamed. The short-listed candidates for the chair were a paediatrician with strong primary health care (PHC) credentials and a physician with a background in health service management and epidemiology. The physician was a hot-blooded 30-something year-old revolutionary recently returned from Oxford University and the powerful influence of Sir Richard Doll, a committed socialist with alleged links to communism! It is unclear why a conservative selection committee gambled on the latter. What follows are some reflections of that now retired revolutionary.

## Context

At the time of my appointment, the political environment was hostile in the aftermath of Steve Biko's death in custody and the 1976 student revolution in Soweto. This resulted in the withdrawal of doctors from many Soweto clinics and the wonderful response of concerned medical practitioners who devoted hours of teaching to the upgrading of clinic-based nurses to function as primary care clinicians in a ‘doctorless’ environment. Nurses ‘specialised’ in either adult or paediatric primary care. However, infrastructure deficiencies and the illogical separation of preventive and curative health care delivery by municipal and provincial authorities challenged both effectiveness and efficiency.

The 1978 Declaration of Alma Ata on PHC set a new gold standard for health care delivery and called for a major shift in health resource allocation, PHC facilities to be the centre piece and the first point of contact between health consumer and health care provider, and the relocation of decision making from a top-down to a bottom-up approach. The PHC approach also called for community participation and inter-sectoral collaboration. This was foreign to and strongly resisted by South Africa's policy-makers.

The Wits medical students were highly politicised, and their leadership was an extraordinary cohort of young people seeking to correct the injustices perpetrated by the apartheid state. They had their own publication *Critical Health* which highlighted health care injustices, and they offered first aid classes to young Sowetans seeking to play their role in the growing revolution. They were supported by a growing number of influential and outraged staff members at the University.

This was the cauldron in which Community Health at Wits was forged. In the decade that followed, the department grew in stature, credibility, and influence despite having few permanent posts, limited resources, having to face setbacks and harassment, and parent institutions whose responses varied from lukewarm tacit support to overt hostility.

## 1980–89

This decade was characterised by carving a niche for academic community health in the hostile corridors of a Medical School steeped in the Oslerian clinical tradition of bedside teaching. There were many who were sympathetic to the need for academic community health but who failed to grasp that its focus was on *population health* and not individual health. The research tools of community health were biostatistics and epidemiology, descriptive and analytical studies culminating in the definitive evidence derived from the double-blind prospective trial with an adequate sample size. These tools were largely foreign to clinicians still wedded to case studies, to clinical intuition and conventional wisdom often derived from bombastic teachers with a compelling stage personality. It is gratifying to see that most clinicians are now fully committed to the hard science of evidence-based medicine as espoused by Cochrane and perfected by a new generation of clinical scientists.

By 1989, the Department of Community Health was the leading publisher, per staff capita, of articles in refereed journals in the Medical School. Wits Community Health provided leadership for a special edition of the *South African Medical Journal* on the Expanded Programme of Immunisation in South Africa – the majority of papers coming from our own staff and students.^1^ The success of this decade rested solely on the passion and quite extraordinary talent and dedication of young students and staff, many in untenured contract posts, supported by loyal administrative personnel for whom no request was unreasonable. Each individual rose to the multiple challenges of academic credibility, research relevance, the inequality of apartheid health care, bringing services to the underserved and unserved sectors of society, health service and health system deficiencies and curriculum reform.

The products of the responses to these challenges included the creation of the Health *Services* (later *Systems*) Development Unit (HSDU), the Centre for Health Policy (CHP), the Wits Rural Facility (WRF), the clinical rural blocks, the Senaoane Health Project in Soweto, the Diploma in Health Service Management to complement the existing Diploma in Public Health, the first comprehensive community health textbook directed at the needs of a developing country, and the establishment of a doctoral student base. Doctoral topics included effectiveness and efficiency of PHC delivery, occupational health hazards notably silicosis and asbestosis, impact of tuberculosis, chronic disability among rural adults, and adult education in the training of PHC nurses. The department prided itself on this wide range of interest and involvement and the consequent growing influence it was to exert on many aspects of health care delivery ranging from policy, through delivery to development to appropriately trained personnel. Another key element of the successes of the 1980s related to the collegiate collaboration amongst like-minded groups at Wits Medical School. We convened ourselves into a *School of Public Health* (Community Health, National Centre for Occupational Health, Family Medicine, Community Dentistry, HSDU, and the CHP incorporating the Women's Health Project). Two departments, Paediatrics and Nursing Education, were supportive but faculty resisted according formal recognition for reasons that never became clear. It was our view that this was to prevent the establishment of a power base calling for curriculum reform, more physical space, and a presence on key faculty structures.

Lack of recognition did not stop us, and we moved from a few offices in the old Colin Gordon Building in Esselen Street, Hillbrow, to the 4th floor at the Parktown Medical School and finally on to the 10th floor where we gradually acquired more and more space. Staff served on the Faculty Executive, the Undergraduate Committee, and the Curriculum Committee. We played seminal roles in the convening of new committees including the Ethics Committee and the Rural Block Committee. Time (3 weeks) for rural block release teaching in the final year of medical studies was negotiated, a visiting support programme of clinical consultants to rural hospitals (Elim, Letaba, and Tintswalo) was arranged, and a permanent presence for Community Health in a rural setting was initiated with the establishment of the HSDU. HSDU evolved into the Health *Systems* Development Unit and spawned the CHP and the WRF, all within this inaugural decade.

Postgraduate training for specialists in community health was a major initiative to expand on the part-time offerings of the diplomas in tropical medicine, public health, and occupational health. To guarantee adequate exposure to the key sub-disciplines meant that service and teaching outlets had to be arranged with relevant stakeholders. These were the National, Provincial, Municipal and Homeland Health Authorities for service exposure, Medical Research Council's Division of Biostatistics and Epidemiology, National Centre for Occupational Health, and Community Health's own academic initiatives for research exposure and other institutions and Faculties for theoretical content, including the South African Institute of Medical Research (SAIMR), the Graduate School of Business and the University Departments of Sociology and Social Anthropology.

The various health service providers ostensibly sponsored registrar posts. Each provider was reluctant to release their appointee for rotation through our various learning experiences, arguing that at all times their employee was accountable to them. This was despite the Academic Head of Community Health enjoying a tri-partite status with *locus standi* in the national and provincial departments and the university. Flexibility to allow rotation was achieved by a generous gesture from the Gazankulu Homeland Health Department agreeing to create two posts for the HDSU, thereby releasing two HSDU posts for the other rotations. The final rotation consisted of 10 appointees rotating between five service sites. Our final challenge was to persuade the usually conservative and traditional South African Medical and Dental Council to recognise a rotation through institutions and experiences previously outside their ambit. Our motivation succeeded and we started what was undeniably the most comprehensive, and what we believed was the most relevant, programme for specialist training in public health in South Africa.

Little of this success would have been possible without generous financial support notably from the Anglo-American Chairman's Fund Educational Trust over many years, Dutch and Swedish Funders, the European Economic Community and American Philanthropic Foundations. Their belief in what we were doing was inspirational and added a further critical dimension to the material support that they gave so generously.

## Postscript

In 2012, as I look back on those early days, I feel that somewhere something went dramatically and almost fatally awry. Was it complacency when the new democratic government was sworn in? Was it sabotage by the old order? Was it the inexperience and lack of skills of the new order? Was it a combination? What was the impact of corruption and misguided expenditure? Had we been naive in advocating universally accessible health care for all South Africans? Had the foundations that were laid been faulty or were they sabotaged? The waters were further muddied by the devastation of the HIV & AIDS pandemic and government denial and failure to endorse primary clinical care nurses as the central pillar of effective health care delivery.

Somehow we got caught up in ideological debates about the need for doctor-centric health care and medical assistants as an alternative. What we had was viable but needed rolling-out. Instead, we had indecision, an action vacuum and a rapid decline in the quality of health care. A burgeoning private health care sector was a distraction. Furthermore, the fear and reluctance of a too-loyal loyal rank and file to engage with and, if necessary, criticise the health leadership, led to wastage and indeed unnecessary deaths. We are all culpable - citizens and health care professionals alike.

This pessimism is offset by the huge body of public health knowledge that has been painstakingly accumulated during the last 15 years, the growth in Public Health at Wits, lessons from the Agincourt Demographic Project and CHP's research over two decades, the great advances in postgraduate offerings, the expansion of interest in rural health care and engagement with local and international policy makers, and most recently, the construction of a building to house the now formally endorsed School of Public Health. The current leadership is up to the task. Mortality statistics are recovering. Changes in the Health Ministry bode well. It is now time for Wits to stand tall in providing ideas, data and evidence that will guide policy formulation, develop human resources, and offer inspiration and critique whether sought or not. The precedent was set in the 1980s; the next decade calls for renewed and redoubled effort.

## Footnotes

1 See *South African Medical Journal*; Volume *72*; Sept 1987.

